# Bacterial and Fungal Coinfection in Critically Ill COVID-19 Cases and Predictive Role of Procalcitonin During the First Wave at an Academic Health Center

**DOI:** 10.1007/s44197-022-00038-4

**Published:** 2022-04-09

**Authors:** Amani M. Alnimr, Mohammed S. Alshahrani, Sara Alwarthan, Shaya Y. AlQahtani, Ahmed A. Hassan, Noor N. BuMurah, Sara Alhajiri, Huda Bukharie

**Affiliations:** 1 Department of Microbiology, College of Medicine, Imam Abdulrahman Bin Faisal University, Dammam, Kingdom of Saudi Arabia; 2 Emergency and Critical Care Department, King Fahad Hospital of the University – Imam Abdulrahman Bin Faisal University, Dammam, Kingdom of Saudi Arabia; 3 Department of Internal Medicine, King Fahad Hospital of the University – Imam Abdulrahman Bin Faisal University, Dammam, Kingdom of Saudi Arabia; 4 Internal Medicine and Critical Care Department, King Fahad Hospital of the University – Imam Abdulrahman Bin Faisal University, Dammam, Kingdom of Saudi Arabia; 5 Department of Critical Care, Faculty of Medicine, Alexandria University, Alexandria, Egypt

**Keywords:** Mortality, Coinfection, SARS-CoV-2, COVID-19, PCT, Biomarkers, Procalcitonin

## Abstract

**Background:**

Coinfection at various sites can complicate the clinical course of coronavirus disease of 2019 (COVID-19) patients leading to worse prognosis and increased mortality. We aimed to investigate the occurrence of coinfection in critically ill COVID-19 cases, and the predictive role of routinely tested biomarkers on admission for mortality.

**Methods:**

This is a retrospective study of all SARS-CoV-2-infected cases, who were admitted to King Fahad Hospital of the University between March 2020 and December 2020. We reviewed the data in the electronic charts in the healthcare information management system including initial presentation, clinical course, radiological and laboratory findings and reported all significant microbiological cultures that indicated antimicrobial therapy. The mortality data were reviewed for severely ill patients who were admitted to critical care units.

**Results:**

Of 1091 admitted patients, there were 70 fatalities (6.4%). 182 COVID-19 persons were admitted to the critical care service, of whom 114 patients (62.6%) survived. The in-hospital mortality was 13.4%. Coinfection was noted in 67/68 non-survivors, and Gram-negative pathogens (*Enterobacterales*, *Pseudomonas aeruginosa*, and *Acinetobacter baumanni)* represented more than 50% of the etiological agents. We noted that the serum procalcitonin on admission was higher for non-survivors (Median = 1.6 ng/mL ± 4.7) than in survivors (Median = 0.2 ng/mL ± 4.2) (*p* ≤ 0.05).

**Conclusion:**

Coinfection is a serious complication for COVID-19 especially in the presence of co-morbidities. High levels of procalcitonin on admission may predict non-survival in critically ill cases in whom bacterial or fungal co-infection is likely.

## Introduction

Severe acute respiratory syndrome coronavirus 2 (SARS-CoV-2) is a novel pathogen implicated in the large coronavirus disease of 2019 (COVID-19) pandemic receiving attention globally. The reported case–fatality rate (CFR) of COVID-19 is substantially heterogenous, and has been estimated between 0.15 and 1% in some studies [1, 2]. Factors that can influence the CFR in case of COVID-19 include the size of undiagnosed asymptomatic or mildly symptomatic cases, geographical location, healthcare system readiness, patient population and co-morbidities [3]. A meta-analysis of 27 age-stratified seroprevalence studies in high-resource settings estimated the CFR of COVID-19 based on age around 0.002, 0.01, 0.4, 1.4, 4.6, 15 and > 25% % at the age of 10, 25, 55, 65, 75, 85, and ≥ 90 years respectively [4].

However, mortality increases in hospitalized patients to around 24% as reported during the early waves in Europe, although it showed a decreasing trend over time [5]. A nation-wide surveillance study in the United Kingdom also showed reduced mortality among critically ill COVID-19 patients from 42 to 20% over a nine-month period, which was attributed to resource allocation [3]. Laboratory predictors of mortality include thrombocytopenia, lymphopenia, elevated biomarkers, such as serum ferritin and inflammatory cytokines like IL6, raised D-dimer or prothrombin time, abnormal liver or renal function tests, and elevated levels of troponin and/or creatine phosphokinase [6, 7]. In addition, there is an increasing body of evidence that high procalcitonin level on presentation may reflect severe illness although it can be normal in many cases of pneumonia [8]. Socioeconomic factors play a major role in the variability of reported in-hospital mortality rates, which are higher in case of resource-limited settings [9–11].

Bacterial and fungal coinfections at different body sites have been described in the initial COVID-19 studies from China in around 8% of the cases, where respiratory and bloodstream infections were the most common [12]. Fungal and bacterial infections have been especially in mechanically ventilated patients [12, 13]. Coinfection with influenza virus and other respiratory pathogens has been reported with a highly variable frequency across the studies [7, 14–18]. The pathogens described were heterogenous and may reflect the differences in testing protocols of COVID-19 patients in different studies. The age was not a significant risk factor for coinfection in one study by Kim et al. [16]. Less frequently recognized pathogens were shown in some cohorts including *Mycoplasma pneumoniae*, *Orientia tsutsugamushi* and dengue virus [18–20]. These pathogens may reflect the local endemicity of diseases and can be underestimated by laboratories that do not routinely perform molecular and serological detection. In our study, we aimed to calculate the in-hospital mortality and describe the clinical course and coinfection among non-survivor, critically ill COVID-19 patients in a university hospital in Saudi Arabia. In addition, the prognostic role of procalcitonin was assessed.

## Materials and Methods

### Research Settings

The retrospective study was conducted at King Fahad Hospital of the University, a 550-bed academic health institution. Cases of SARS-CoV-2 infection admitted to our hospital during the first wave of the pandemic in Saudi Arabia, between March 2020 and December 2020. We excluded asymptomatic watchers for patients who were detected by routine screening upon admission, cases diagnosed elsewhere and referred to us in a late status, and dead cases on arrival to the hospital. All cases suspected to have COVID-19 followed a respiratory pathway on presentation and throughout the admission. The data in the electronic charts in the healthcare information management system were systematically reviewed including initial presentation, clinical course, radiological and laboratory findings with significant microbiological cultures that indicated antimicrobial therapy. Coinfection was defined by significant growth of representative cultures in patients with clinical or radiological deterioration that indicated sample collection.

The severity of illness among COVID-19 confirmed patients was clinically determined based on the local national protocol [21]. The mortality-related data were collected for severely ill patients who were admitted to critical care units. Approval of the Institutional Review Board was obtained (IRB-2020-01-150).

### Viral Assays

Nasopharyngeal swabs with viral transport media (Vircell, Granada, Spain) were used to screen for SARS-CoV-2 on admission and transported on ice pack to the laboratory for immediate processing. Reverse transcriptase polymerase chain reaction (RT-PCR) targeting SARS-CoV-2 was performed using the Xpert^®^ Xpress SARS-CoV-2 (Cepheid, Sunnyvale, United States), which identifies the *E* gene and *N2* gene of SARS-CoV-2 following the manufacturer’s recommendations and using positive and negative controls. All samples were processed under a biosafety cabinet Class II Type B to minimize the occupational risk, in a biosafety level 2 laboratory as no viral propagation or aerosol-generating procedure was performed.

### Bacterial and Yeast Identification

Representative clinical specimens from the suspected sites of infections (sputum, tracheal aspirates, urine, soft tissues surrounding the vascular lines) were grown on the corresponding routine bacteriological media including Columbia Blood agar, chocolate agar, MacConkey agar plates (SPML, Riyadh, Saudi Arabia), and were incubated aerobically and anaerobically at 35 °C for 24–48 h. Any significant growth for the site was identified using the VITEK^®^ MS (bioMe ´rieux Inc., Durham, NC, USA), an automated mass spectrometry microbial identification system based on matrix-assisted laser desorption ionization time-of-flight (MALDI-TOF) technology. Antimicrobial susceptibility testing was performed by VITEK 2 automated system (bioMe ´rieux Inc., Durham, NC, USA) as per the manufacturer’s recommendations.

### Procalcitonin Measurement

The ADVIA Centaur^®^ XPT Immunoassay System (Siemens, Munich, Germany) was utilized following the manufacturer’s instructions to measure quantitative levels of procalcitonin on admission as a part of the hospital policy for all cases suspected to have SARS-CoV-2 infection.

### Statistical Analysis

The statistical analysis was performed using the Graphpad Prism Version 9.0. Kolmogorov–Smirnov test was measured to assess the difference of procalcitonin analyte levels between survivors and non-survivors. A significance value of *p* ≤ 0.05 was used.

## Results

**Fig. 1:**
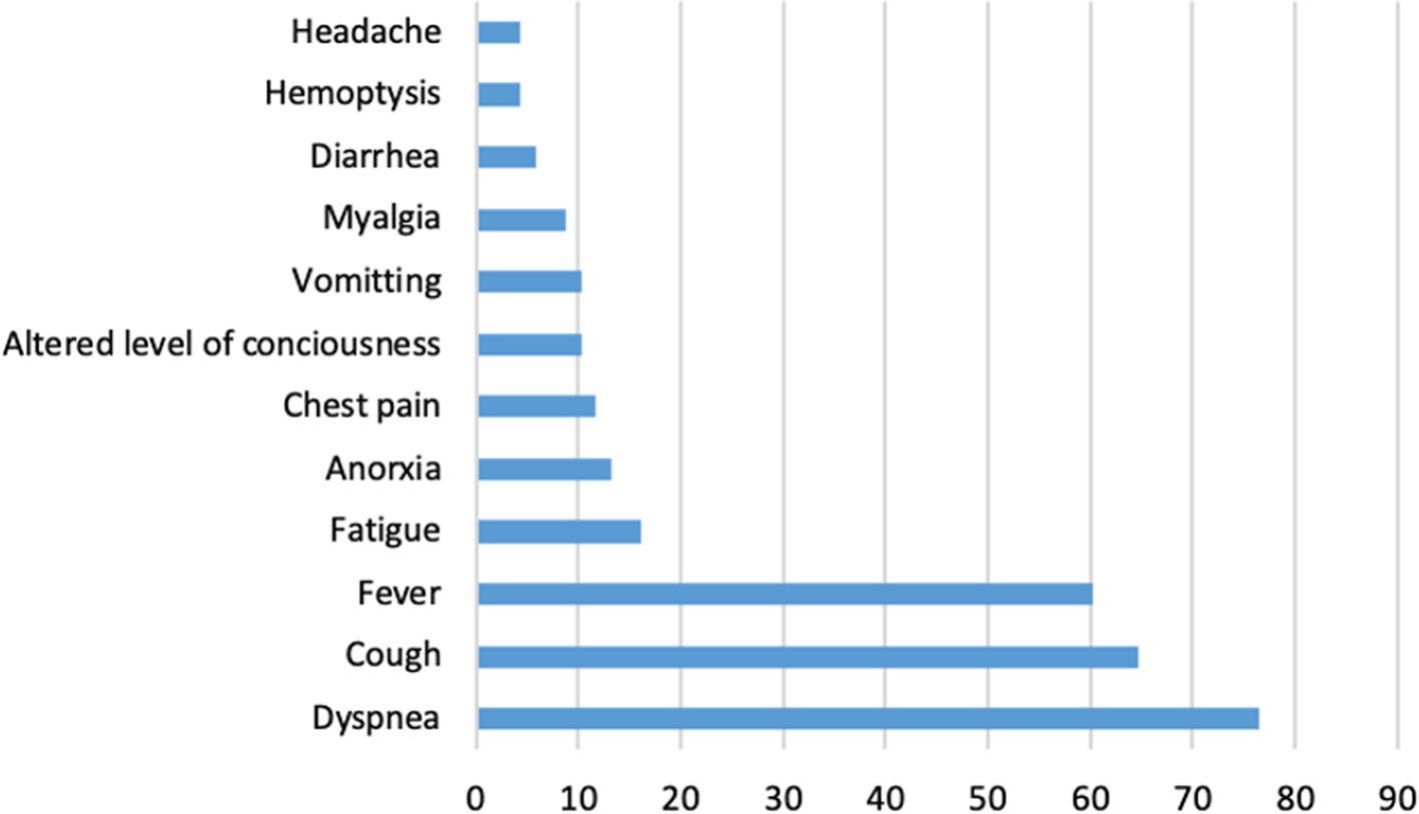
Initial clinical presentation of the 68 non-survivor COVID-19 patients

A total of 1091 patients were diagnosed, among whom there were 70 fatalities (6.4%). The median age for all patients was 48 years ± 4.3. The total number of COVID-19 cases who required admission was 521, with 70 deaths (in-hospital mortality rate = 13.4%). 182 cases required critical care admission, of whom 114 patients (62.6%) survived and 68 (37.4%) passed away. The non-survivors had a median body mass index (BMI) of 26.9 ± 3.1 and were 14 females (20.6%) and 54 (79.4%) males of whom 26 cases (38.3%) were Saudi and 42 (61.7%) were non-Saudi. The total case fatality rates in Saudi and non-Saudi patients were 28/639 (4.4%) and 42/452 (9.3%) (Odds Ratio = 2.1 95% CI 1.2–2.9). Figure 1 and Tables 1 and 2 summarize the initial clinical presentation for the 68 non-survivor patients.

**Table 1:** Initial clinical findings of 68 non-survivor COVID-19 patients

Parameter	Median (range)
Temperature	37.5 C (36.1–40.2)
Respiratory rate	28.4 per min (18–54)
O_2_ saturation	82.3% (43–100)
Heart rate	101 per min (62–150)
Mean arterial pressure	90 mmHg (60.3–161)

**Table 2:** Radiological findings of 68 non-survivor COVID-19 patients

Radiological findings	At presentation	Upon intubation
Bilateral infiltrate	55 (80.9%)	62 (91.2%)
Unilateral infiltrate	4 (5.9%)	1 (1.5%)
Pulmonary embolism	3 (4.4%)	1 (1.5%)
Pneumothorax	–	3 (4.4%)
Pneumopericardium	–	1 (1.5%)
No abnormality detected	6 (8.8%)	–

Mortality was highest for patients aged 60 years or older; 47/70 (67.1%), followed by middle-aged cases between 31 and 59 years old 23/70 (32.9%). No mortality was encountered in patients between 0 and 30 years old.

The median time to intubation for the non-survivors was 4 days (0–32), with a median duration of intubation around 11 days (0–38). Invasive mechanical ventilation was used in 63/68 (92.6%) patients while non-invasive mechanical ventilation or oxygen supplementation were used in 36 (52.9%) and 40 (58.8%) patients respectively (Table 3).

**Table 3:** Comorbidities in 68 non-survivor COVID-19 patients

Condition	Number of cases (%)
Diabetes mellitus	20 (29.4%)
Hypertension	21 (30.9%)
Congestive heart failure	5 (7.4%)
End stage renal disease	10 (14.7%)
Malignancy	2 (2.9%)
Obstructive lung disease	6 (8.8%)
Cerebrovascular accident	5 (7.4%)
Coronary artery diseases	13 (19.1%)
Anemia	4 (5.9%)
Chronic liver disease	8 (11.8%)
Smoking	15 (22.1%)

Bacterial and fungal coinfections were noted among all but one non-survivor (67/68). Table 4 outlines the types of infection and pathogens identified in those cases. We noted that the serum procalcitonin on admission was higher for non-survivors (Median = 1.6 ng/mL ± 4.7) than in survivors (Median = 0.2 ng/mL ± 4.2) (*p* ≤ 0.05) as evident in Fig. 4. Other laboratory findings for the non-survivor group exhibited abnormal renal function tests in 32 (47.1%) raised LDH in 61 (89.7%), lymphopenia in 39 (57.4%), and neutropenia in only 1 case (1.5%).

**Table 4:** Frequency of coinfections diagnosed in 67 non-survivor cases of COVID-19 between March and December 2020

Number of cases	Bloodstream infection	Respiratory infections	Any other site infection	Multiple site infections	Total (%)
*Staphylococcus aureus*	2	4	–	1	7 (10.3%)
Other *Streptococci*	2	–	–	–	2 (2.9%)
*Enterococcus* spp.	1	–	1	1	3 (4.4%)
*E. coli*	–	1	–	–	1 (1.5%)
*Klebsiella* spp.	–	7	–	–	7 (10.3%)
*Enterobacter* spp.	1	–	–	–	1 (1.5%)
*Pseudomonas aeruginosa*	2	8	1	2	13 (19.1%)
*Acinetobacter baumannii*	3	9	–	1	13 (19.1%)
*Candida* spp.	4	–	3	–	7 (10.3%)
*Aspergillus* spp.	–	1	–	–	1 (1.5%)
Other pathogens	–	3	1	–	4 (5.8%)
Polymicrobial infections	2	2	4	–	8 (11.8%)
Total	17 (25%)	39 (57.4%)	6 (8.8%)	5 (7.4%)	67 (98.6%)

## Discussion

This study included all confirmed COVID-19 cases in an academic health institution with an overall case fatality rate of 6.4% during the first wave prior to the introduction of the COVID-19 vaccines. This rate is lower than in-hospital fatality in several countries [3, 5], which be attributed to several explanation, including the younger local patient population, healthcare infrastructure and virus-related factors. Nevertheless, the higher case fatality rate seen among other ethnic groups in our institution (Odds Ratio = 2.1 95% CI 1.2–2.9) raises a concern. Whether this phenomenon is related to host immune factors or social determinants leading to delayed presentation is unclear and worth further investigation. As in other studies, the mortality was increased proportionally with age in the described cohort [3].

**Fig. 2:**
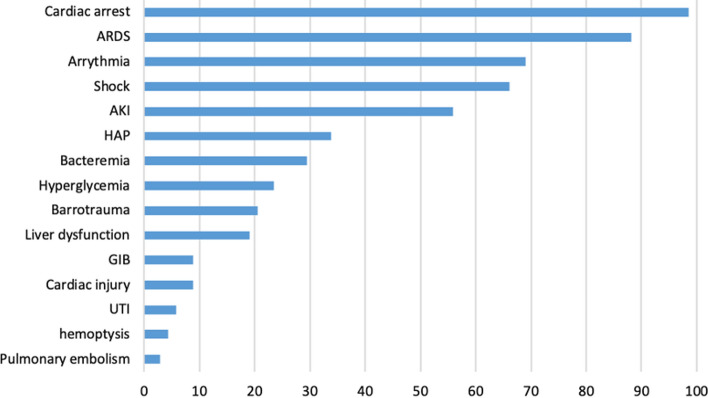
Complications in the clinical course of 68 non-survivor COVID-19 patients. *ARDS* Acute respiratory distress syndrome, *AKI* acute kidney injury, *HAP* hospital−acquired pneumonia, *GIB* Guillain–Barre syndrome, *UTI* urinary tract infection

**Fig. 3:**
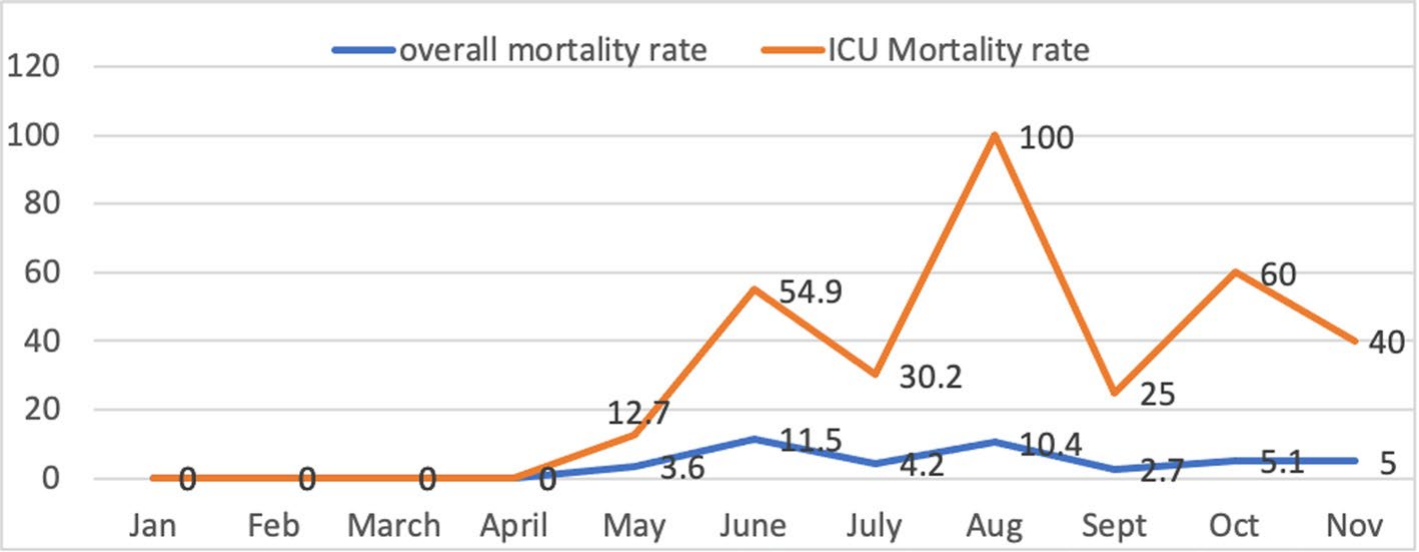
Mortality rate across the first wave of COVID-19 pandemic in 2020

Furthermore, the complications encountered in the described patient population were previously described, such as barotrauma, pneumomediastinum, bacteremia and other infections [22]. In our cohort, coinfection was a frequent event in non-survivors and Gram-negative pathogens, namely *Enterobacterales*, *Pseudomonas aeruginosa*, and *Acinetobacter baumanni*, represented more than 50% of the etiological agents. The predominance of Gram-negative pathogens in our cohort can be interpreted by the inhabitance of hospital environments by these organisms which are known to harbor multidrug-resistance determinants adding to the complexity of management of COVID-19 cases [23]. Some studies reported lower incidence of coinfection because the study was designed to include co-infections in all COVID-19 patients, whereas our data were restricted to non-survivors [13, 24]. It is difficult to accurately estimate coinfection rate in all COVID-19 cases due to the fact that not all patients are being tested serially for coinfection, particularly during the early phase of the COVID-19 pandemic due to pressure on the healthcare systems. In a secondary-care study performed in England, 77% of COVID-19 cases had blood culture but 15% only had respiratory culture work-up, which can explain the heterogeneity in confection rates across the literature, although it is difficult to ascertain the true infection from colonization in some of those respiratory cultures [25]. Based on combined microbiological and clinical data, 25% and 57.4% of the non-survivors in our institution had bloodstream or respiratory infection respectively with 7.4% developing multiple infections mainly lower respiratory infections and bacteremia (Table 4). Post-viral bacterial pneumonia is thought to be mediated by complex interactions with the nasopharyngeal flora and the host immune system [26]. It was previously shown that influenza virus markedly reduced tracheal mucus velocity during the early phase of infection that continued for up to several weeks [27]. Thus, upper airway flora gets access to the lung parenchyma leading to secondary bacterial infections. Coinfection by influenza virus and other respiratory viruses in COVID-19 patients has been also described [14]. In our testing protocol, only influenza virus was routinely included and we did not detect any influenza coinfection among the first wave of COVID-19 cases (Figs. 2, 3).

**Fig. 4:**
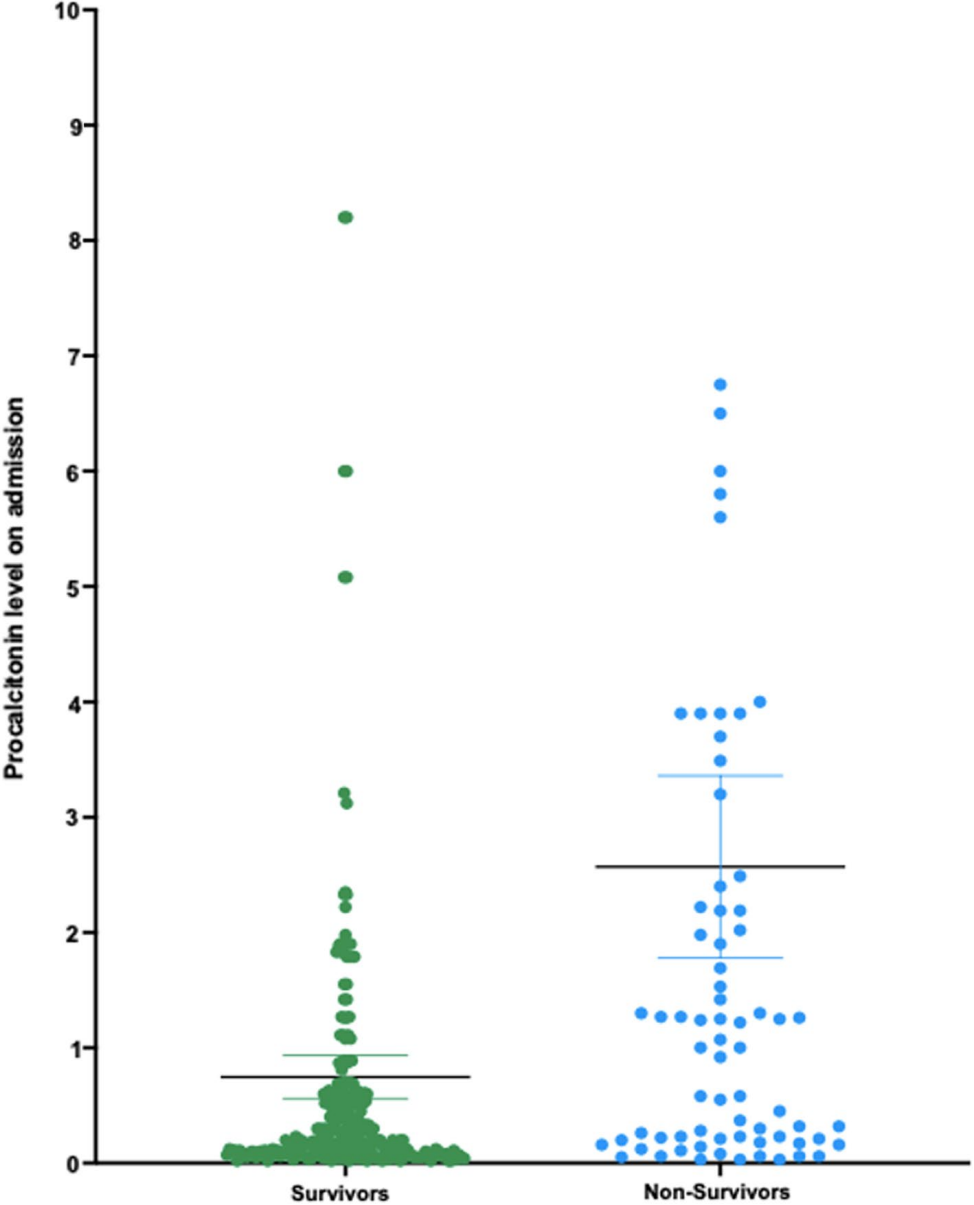
Procalcitonin levels in non-survivor COVID-19 patients in comparison to survivors

Nearly all non-survivor patients in our study received mechanical ventilation. A previous study has shown 60% mortality among mechanically ventilated patients [5]. Among the potential prognostic factors in COVID-19 cases is the initial serum level of procalcitonin which may reflect disease severity [28, 29]. Our findings are consistent with a study by Liu et al. whose work suggested that mortality can be inferred from early measurements of procalcitonin levels (Fig. 4) [30]. Krause et al. have also found an association between early procalcitonin level > 0.1 ng/ml and duration of mechanical ventilation [31]. Procalcitonin has been used as a stewardship tool to distinguish bacterial from non-bacterial etiologies of inflammation especially in cases of lower respiratory tract infections [32]. Thus, high initial procalcitonin may reflect early bacterial coinfection in those cases since viral infections facilitate invasiveness by upper airways commensal opportunistic flora. Nevertheless, the rise in procalcitonin may be related to generic systemic inflammation seen in the later stages of illness that is not specific for secondary infections and so this phenomenon needs to be further examined [33]. Other promising prognostic markers in COVID-19 cases are under assessment, such as the neutrophil-to-lymphocyte ratio, although they are in need of further evaluation [34].

The main limitation of this study is its reliance on date representing a specific institutional experience rather than being a population-level study. In addition, the retrospective nature of the study did not allow standardizing the times of microbiological testing to investigate coinfection. Nevertheless, it contributes to the growing evidence of variable post-acute sequelae of the illness and mortality rates in relation to coinfections and other factors that represent challenges during the clinical course of COVID-19 infection.

## Conclusion

This study showed that the in-hospital COVID-19 mortality is lower in case of high-resource settings and younger patient population. It also highlighted coinfection as a serious complication for SARS-CoV-2 leading to fatality, particularly in patients with other co-morbidities. High initial levels of procalcitonin on presentation may predict poor prognosis in critically ill patients who require admission to intensive care.

## Data Availability

No supplementary files are enclosed. Raw data can be submitted on a justifiable request.

## Abbreviations

CFR: Case–fatality rate (CFR)
LDH: Lactate dehydrogenase
RT-PCR: Reverse transcriptase polymerase chain reaction
SARS-CoV-2: Severe acute respiratory syndrome coronavirus 2
